# Submucosal Gastric Mass Mimicking GIST: Final Diagnosis of Vanek’s Tumor

**DOI:** 10.3390/diagnostics16132035

**Published:** 2026-06-29

**Authors:** Ljubica Lazic, Milica Mitrovic, Anja Zugic, Zeljko Grubac, Katarina M. Eric, Nenad Ivanovic, Aleksandra Djuric-Stefanovic, Ognjan Skrobic, Keramatollah Ebrahimi

**Affiliations:** 1 Center for Radiology and Magnetic Resonance Imaging, University Clinical Centre of Serbia, Pasterova No. 2, 11000 Belgrade, Serbia; ljbc.lazic@gmail.com (L.L.); aleksandra.djuricstefanovic@gmail.com (A.D.-S.); 2 Faculty of Medicine, University of Belgrade, Dr Subotica No. 8, 11000 Belgrade, Serbiaskrobico@gmail.com (O.S.); keramatollahe@yahoo.com (K.E.); 3 Clinic for Digestive Surgery, University Clinical Centre of Serbia, Koste Todorovica Street, No. 6, 11000 Belgrade, Serbia; anja.zugic@gmail.com (A.Z.); zeg18@hotmail.com (Z.G.); 4 Department of Pathology, First University Surgical Clinic, University Clinical Centre of Serbia, Koste Todorovica Street, No. 6, 11000 Belgrade, Serbia; ketrineric@gmail.com

**Keywords:** IFP, Vanek’s tumor, GIST, submucosal lesions, gastric tumor

## Abstract

Inflammatory fibroid polyp (IFP) or Vanek’s tumor is a rare benign submucosal lesion of the gastrointestinal tract that may radiologically mimic mesenchymal gastric tumors, particularly gastrointestinal stromal tumors. We present the case of a 65-year-old patient with a contrast-enhancing gastric submucosal mass detected on computed tomography, initially interpreted as a suspected mesenchymal neoplasm. CT imaging demonstrated a well-defined enhancing lesion arising from the gastric wall without evidence of metastatic disease. Surgical resection was performed because imaging findings were considered highly suggestive of GIST. Gross intraoperative appearance and pathological examination, however, established the final diagnosis of gastric inflammatory fibroid polyp (Vanek’s tumor). Histopathological analysis demonstrated characteristic spindle-cell proliferation with inflammatory eosinophil-rich infiltrates, while immunohistochemistry excluded GIST. This case highlights the diagnostic challenge in differentiating IFP from other gastric submucosal neoplasms based solely on imaging findings and emphasizes the importance of histopathological confirmation for definitive diagnosis.

**Figure 1:**
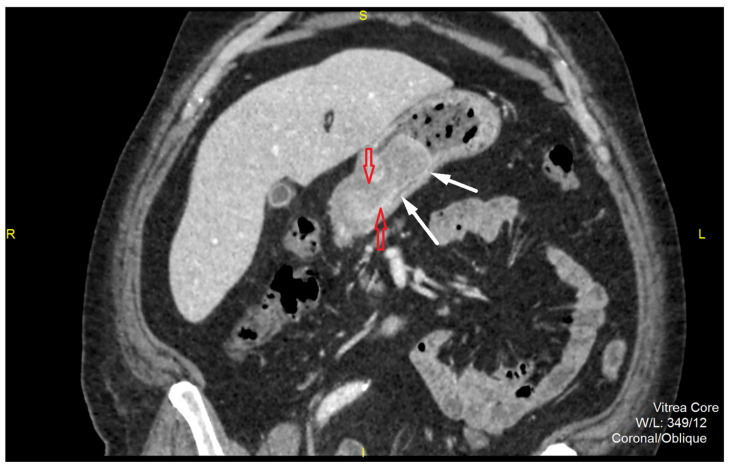
Contrast-enhanced CT showing a well-circumscribed enhancing intraluminal prepyloric gastric mass (white arrows) measuring 35 × 40 × 60 mm. The lesion demonstrates homogeneous enhancement without necrosis, cystic degeneration, or calcification, and the overlying mucosa appears preserved. Limited gastric distension due to patient intolerance hindered complete evaluation of the lesion attachment (red arrows).

**Figure 2:**
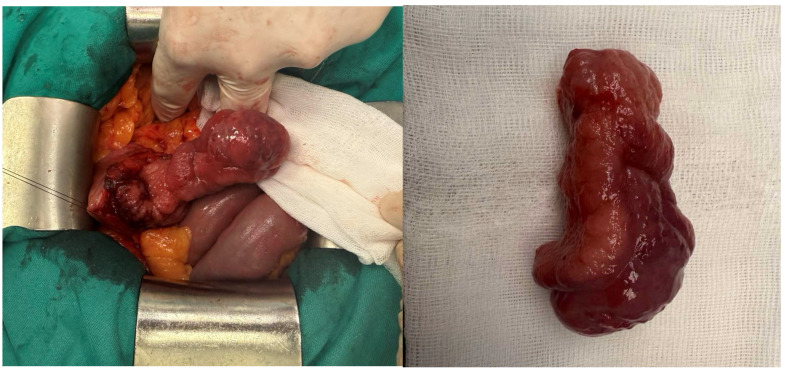
Intraoperatively, a well-circumscribed intraluminal polypoid lesion was identified arising from the gastric wall. The lesion demonstrated a pedunculated configuration without evidence of transmural invasion or involvement of adjacent structures. Complete resection with negative surgical margins was achieved.

**Figure 3:**
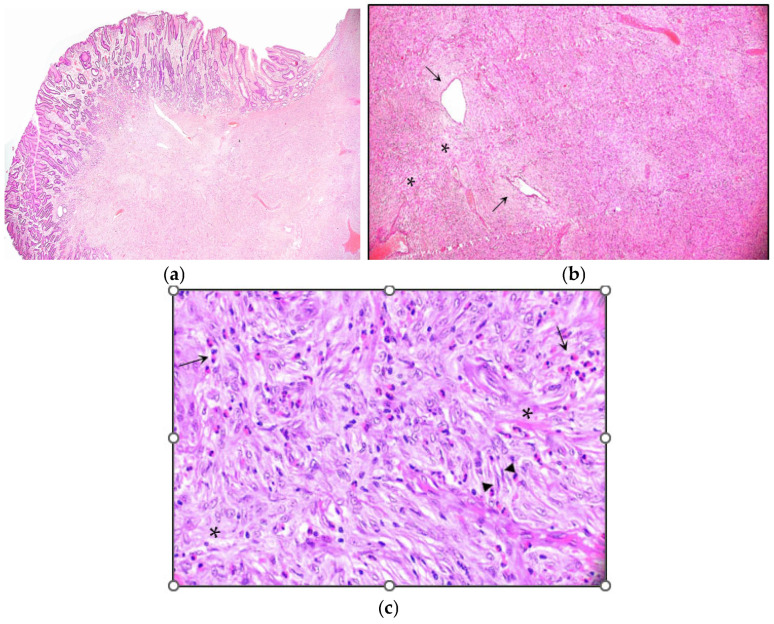
Histopathological findings of inflammatory fibroid polyp (Vanek’s tumor): (**a**) submucosal lesion covered by intact foveolar mucosa (H&E, ×20); (**b**) spindle-cell proliferation (arrowheads) with concentric perivascular (“onion-skin”) arrangement (H&E, ×40); (**c**) eosinophil-rich inflammatory infiltrate (black arrows)within a fibromyxoid stroma (H&E, ×400, asterisks). Immunohistochemical analysis excluded Gastrointestinal stromal tumor by the absence of typical GIST immunophenotypic markers, including CD117 and DOG1 expression [1]. The final diagnosis was therefore established as a gastric inflammatory fibroid polyp (Vanek’s tumor). Gastrointestinal stromal tumor represents the most common mesenchymal tumor of the gastrointestinal tract and originates from the interstitial cells of Cajal [1]. Gastric GISTs usually present as subepithelial masses with sessile or exophytic growth and variable enhancement patterns depending on tumor size, vascularity, and the presence of necrosis or hemorrhage [1,2]. In contrast, IFP is a rare benign submucosal lesion characterized histologically by spindle-cell proliferation accompanied by eosinophil-rich inflammatory infiltrates [3,4]. Because IFPs may appear on CT as enhancing intraluminal submucosal masses, differentiation from GIST based solely on imaging findings may be difficult, particularly in cases with inadequate gastric distension and limited visualization of lesion morphology [2,3,4]. In addition to GIST, the differential diagnosis of an enhancing gastric submucosal mass includes leiomyoma, schwannoma, ectopic pancreas, and other subepithelial lesions, many of which may exhibit overlapping radiological and endoscopic characteristics, making preoperative diagnosis challenging. While CT provides valuable information regarding lesion size, enhancement pattern, and possible extragastric extension, endoscopy and EUS allow more precise assessment of mucosal involvement, layer of origin, attachment pattern, and internal architecture. In particular, EUS may help distinguish benign polypoid lesions, including inflammatory fibroid polyps, from mesenchymal tumors such as GIST, although histopathological confirmation remains necessary in many cases. Therefore, accurate characterization of gastric submucosal lesions is best achieved through a multimodality approach combining CT, endoscopic evaluation, EUS when available, and histopathological analysis [5]. A limitation of this report is that the upper gastrointestinal endoscopy was performed at an outside institution, and the original endoscopic images were not available for inclusion in this manuscript. The present case is consistent with previous reports describing gastric inflammatory fibroid polyp, or Vanek’s tumor, as a rare benign submucosal lesion that most commonly occurs in the gastric antrum and may clinically and radiologically simulate a Gastrointestinal stromal tumor [3,4]. Similar to the case reported by Silva et al., in which a gastric inflammatory fibroid polyp was initially suspected to represent GIST, our lesion was interpreted preoperatively as a probable mesenchymal gastric tumor because of its enhancing submucosal appearance [6]. Retrospective image analysis suggested a pedunculated configuration, a feature that may favor a benign polypoid lesion rather than a typical mesenchymal neoplasm. Several authors have emphasized that preoperative diagnosis of gastric inflammatory fibroid polyps is difficult, since endoscopic biopsies may be non-diagnostic and cross-sectional imaging lacks sufficiently specific features to reliably distinguish it from GIST or other subepithelial tumors [6,7]. In our case, this diagnostic uncertainty was further increased by suboptimal gastric distension on CT, which limited precise assessment of the lesion attachment. Inadequate gastric distension obscured pedunculated morphology and contributed to preoperative misclassification as GIST. Retrospectively, the pedunculated intraluminal morphology represented an important clue favoring a benign polypoid lesion rather than a typical GIST, which more often presents as a sessile subepithelial mass with intramural or exophytic growth [1,2,8]. The therapeutic implication of this distinction is clinically relevant. While localized gastric GIST is generally managed by complete surgical resection with negative margins, inflammatory fibroid polyps are benign lesions and, when technically feasible, may be treated by less invasive endoscopic resection [1,7,9]. Inayat et al. and recent reviews have highlighted the increasing role of endoscopic resection for gastric inflammatory fibroid polyps, although surgery remains appropriate for larger lesions, uncertain diagnosis, or technically challenging cases [7,9]. Based on the combination of endoscopic morphology and the presumptive diagnosis of GIST, a multidisciplinary decision was made to proceed with surgical wedge resection. In our patient, wedge resection was selected because the preoperative impression favored GIST; however, if Vanek’s tumor had been suspected with greater confidence, endoscopic polypectomy or endoscopic submucosal dissection might have been considered. In our case endoscopic resection was not considered appropriate because of the wide attachment of the lesion and the perceived risk of serosal injury or perforation. The choice between endoscopic and surgical resection of gastric submucosal lesions depends on lesion size, morphology, layer of origin, technical feasibility, and the degree of suspicion for malignancy. Endoscopic techniques, including endoscopic mucosal resection (EMR) and endoscopic submucosal dissection (ESD), are generally preferred for lesions with a favorable intraluminal configuration and a low risk of perforation, whereas surgical resection remains the standard approach for lesions suspected to represent GIST or those considered technically challenging for endoscopic removal. In the present case, preoperative endoscopy performed at an outside institution described a broad-based lesion, raising concerns regarding the risk of serosal injury or perforation during endoscopic resection. Furthermore, CT findings strongly favored a mesenchymal tumor, resulting in a multidisciplinary decision to proceed with wedge resection. Although the lesion exhibited predominantly intraluminal growth, preoperative suspicion of an inflammatory fibroid polyp might have prompted consideration of an endoscopic approach; however, the broad attachment base would still have represented an important technical limitation [7,9]. Our case supports previous observations that Vanek’s tumor should be included in the differential diagnosis of an enhancing gastric submucosal mass, particularly when the lesion shows an intraluminal polypoid or pedunculated configuration. Adequate gastric distension during CT examination is essential, as poor luminal distension may obscure the stalk and lead to overestimation of a mesenchymal tumor pattern.

## Data Availability

The datasets used and analyzed in this study are available from the corresponding author upon reasonable request.

## References

[1] Miettinen M., Lasota J. (2006). Gastrointestinal stromal tumors: Pathology and prognosis at different sites. Semin. Diagn. Pathol..

[2] Nonose R., Valenciano J.S., da Silva C.M.G., Silva R.C., de Oliveira A.P., Fernandes M.T., Costa L.F., Pinto J.C., Barros M.A., Santos F.P. (2011). Gastrointestinal stromal tumors: Imaging findings. Radiol. Bras..

[3] Johnstone J.M., Morson B.C. (1978). Inflammatory fibroid polyp of the gastrointestinal tract. Histopathology.

[4] Daum O., Hes O., Vanecek T., Benes Z., Sima R., Zamecnik M., Mukensnabl P., Hadravska S., Curik R., Michal M. (2003). Vanek’s tumor (inflammatory fibroid polyp). Report of 18 cases and comparison with gastrointestinal stromal tumors. Virchows Arch..

[5] Nishida T., Kawai N., Yamaguchi S., Nishida Y. (2013). Submucosal tumors: Comprehensive guide for the diagnosis and therapy of gastrointestinal submucosal tumors. Dig. Endosc..

[6] Silva M., Albuquerque A., Cardoso H., Costa J., Macedo G. (2016). Gastric inflammatory fibroid polyp mimicking a gastrointestinal stromal tumor. Rev. Esp. Enferm. Dig..

[7] Garmpis N., Damaskos C., Garmpi A., Georgakopoulou V.E., Sakellariou S., Liakea A., Schizas D., Diamantis E., Farmaki P., Voutyritsa E. (2021). Inflammatory fibroid polyp of the gastrointestinal tract: A systematic review for a benign tumor. In Vivo.

[8] Kawai A., Matsumoto H., Haruma K., Kanzaki T., Sugawara Y., Akiyama T., Hirai T. (2020). Rare case of gastric inflammatory fibroid polyp located at the fornix mimicking gastric cancer. Surg. Case Rep..

[9] Inayat F., Ur Rahman A., Wahab A., Riaz A., Zahid E., Bejarano P., Pimentel R. (2020). Gastric inflammatory fibroid polyp: A rare cause of occult upper gastrointestinal bleeding. Cureus.

